# Selective Phosphodiesterase 1 Inhibitor BTTQ Reduces Blood Pressure in Spontaneously Hypertensive and Dahl Salt Sensitive Rats: Role of Peripheral Vasodilation

**DOI:** 10.3389/fphys.2020.543727

**Published:** 2020-09-08

**Authors:** Asim B. Dey, Sherif Khedr, James Bean, Leah L. Porras, Tamika D. Meredith, Francis S. Willard, Joseph V. Hass, Xin Zhou, Maia Terashvili, Cynthia D. Jesudason, Kevin M. Ruley, Michael R. Wiley, Mark Kowala, Simon J. Atkinson, Alexander Staruschenko, Mark D. Rekhter

**Affiliations:** ^1^Eli Lilly and Company, Indianapolis, IN, United States; ^2^Department of Physiology, Medical College of Wisconsin, Milwaukee, WI, United States; ^3^Department of Physiology, Faculty of Medicine, Ain Shams University, Cairo, Egypt; ^4^Department of Biology, Indiana University – Purdue University Indianapolis, Indianapolis, IN, United States; ^5^Clement J. Zablocki VA Medical Center, Milwaukee, WI, United States

**Keywords:** phosphodiesterase 1, arterial hypertension, spontaneously hypertensive rat, Dahl salt sensitive rat, vasodilation

## Abstract

Regulation of the peripheral vascular resistance via modulating the vessel diameter has been considered as a main determinant of the arterial blood pressure. Phosphodiesterase enzymes (PDE1-11) hydrolyse cyclic nucleotides, which are key players controlling the vessel diameter and, thus, peripheral resistance. Here, we have tested and reported the effects of a novel selective PDE1 inhibitor (BTTQ) on the cardiovascular system. Normal Sprague Dawley, spontaneously hypertensive (SHR), and Dahl salt-sensitive rats were used to test *in vivo* the efficacy of the compound. Phosphodiesterase radiometric enzyme assay revealed that BTTQ inhibited all three isoforms of PDE1 in nanomolar concentration, while micromolar concentrations were needed to induce effective inhibition for other PDEs. The myography study conducted on mesenteric arteries revealed a potent vasodilatory effect of the drug, which was confirmed *in vivo* by an increase in the blood flow in the rat ear arteriols reflected by the rise in the temperature. Furthermore, BTTQ proved a high efficacy in lowering the blood pressure about 9, 36, and 24 mmHg in normal Sprague Dawley, SHR and, Dahl salt-sensitive rats, respectively, compared to the vehicle-treated group. Moreover, additional blood pressure lowering of about 22 mmHg could be achieved when BTTQ was administered on top of ACE inhibitor lisinopril, a current standard of care in the treatment of hypertension. Therefore, PDE1 inhibition induced efficient vasodilation that was accompanied by a significant reduction of blood pressure in different hypertensive rat models. Administration of BTTQ was also associated with increased heart rate in both models of hypertension as well as in the normotensive rats. Thus, PDE1 appears to be an attractive therapeutic target for the treatment of resistant hypertension, while tachycardia needs to be addressed by further compound structural optimization.

## Introduction

Despite the significant improvement in patient care, resistant arterial hypertension remains an unmet medical need (Carey et al., 2018). A number of drugs, including compounds affecting renin-angiotensin-aldosterone system (RAAS), diuretics, beta-blockers, and calcium channel blockers are currently in practice; however, current therapeutic approaches are still limited (Kitt et al., 2019). It is feasible that the drugs targeting RAAS are missing critical RAAS-independent mechanistic nodes. Specifically, vascular smooth muscle cell (SMC) contractility, the focal point defining peripheral vascular resistance, is likely to be partially regulated in RAAS-independent manner (Muñoz-Durango et al., 2016).

Smooth muscle cell relaxation is primarily mediated by 3′, 5′-cyclic guanosine monophosphate (cGMP). cGMP level is determined by the balance between the rate of synthesis, mediated by guanylate cyclases, and degradation, through cyclic nucleotide phosphodiesterases (PDEs). The superfamily of PDEs consists of 11 PDE families (PDE1–PDE11) formed by multiple genes and isoforms. The role of PDE5 and PDE3 is widely explored in this context (Turko et al., 1999; Bellamy and Garthwaite, 2001; Corbin, 2004). PDE1 is highly expressed in SMCs (Giachini et al., 2011; Khammy et al., 2017). However, much less attention has been paid to PDE1. There are three isoforms of PDE1: A, B, and C. PDE1A and PDE1B have a substrate preference for cGMP, and PDE1C has equal affinity for cGMP and 3′, 5′-cyclic adenosine monophosphate (cAMP). PDE1 isozymes are unique among PDEs, in that they are activated by calcium-calmodulin (Maurice et al., 2014), and this may be critical in the context of hypertension featuring enhanced calcium signaling (Pinterova et al., 2011). Finally, genome-wide association studies (GWAS) have reported an association between PDE1A snp and blood pressure (Tragante et al., 2014; Bautista Nino et al., 2015). While the role of PDE1 in vascular reactivity has been highlighted in several papers, systematic research employing potent and selective PDE1 inhibitors in animal models of hypertension is lacking.

In this manuscript, we presented a novel and potent PDE1 inhibitor BTTQ that is highly selective for PDE1 over all other phosphodiesterases, but equipotent for all isoforms of PDE1 (A, B and C). This compound possesses absorption, distribution, metabolism, excretion (ADME) characteristics, allowing its use *in vivo* (Figure 2C and Supplementary Table 3). BTTQ demonstrated blood pressure (BP) lowering effects in both normotensive rats and animals with hypertension of different genesis (SHR, a high renin model, and Dahl salt-sensitive hypertension, a low renin model) suggesting the pattern of activity independent of the primary mechanisms of hypertension. Moreover, additional BP lowering could be achieved when BTTQ was administered on top of ACE inhibitor lisinopril, a current standard of care in the treatment of hypertension. These anti-hypertensive effects are likely associated with vasodilatory properties of BTTQ that we have demonstrated *in vitro*, using isolated mesenteric arteries, and *in vivo*, using novel PK/PD model.

## Materials and Methods

### Animals

The animal care and experimental protocols in this study were conducted under the supervision of a veterinarian following a protocol reviewed and approved by the Eli Lilly and Company’s Animal Care and Use Committees or by the IACUC at the Medical College of Wisconsin. The procedures in this protocol are in compliance with the U.S. Department of Agriculture’s (USDA) Animal Welfare Act (9 CFR Parts 1, 2, and 3); the Guide for the Care and Use of Laboratory Animals (Institute for Laboratory Animal Research, The National Academies Press, Washington, DC, United States); and the National Institutes of Health, Office of Laboratory Animal Welfare (for NIH funded studies). Whenever possible, procedures in this study are designed to avoid or minimize discomfort, distress, and pain to animals. All the rats used in this study were between 6 and 8 weeks for the SD, SHR, and Dahl Salt sensitive rats studies.

### Generation of PDE Proteins

All PDE proteins were expressed in baculoviral infected insect cells using standard methods [pFastBac, pFastBac Dual (Invitrogen), pIEX4 (Novagen)]. See Supplementary Table 1 for DNA sequences, construct boundaries, affinity tags, and expression vectors. HIS_6_ tagged PDE proteins were purified using Ni-NTA agarose (Qiagen) followed by size exclusion chromatography on a Superdex 200 column (GE Healthcare) in storage buffer (20 mM Tris-HCl, pH 7.5, 150 mM NaCl, 10% Glycerol). FLAG-tagged PDE proteins including the PDE6A/6B heterodimer were purified using anti-FLAG M2-agarose (Sigma-Aldrich), after purification through NiNTA column chromatography and eluted in storage buffer (50 mM Tris-HCl, pH 7.5, 150 mM NaCl, 10% Glycerol, 0.1 mg/ml Flag peptide). All purified proteins were stored at −80°C in small aliquots.

### Phosphodiesterase Enzyme Assays

All PDE enzyme activities were measured with a yittrium silicate based scintillation proximity assay that detects radioactive nucleotide monophosphates but not cyclic monophosphates. Assays are conducted in a total volume of 50 μl in 384 well plates (3706, Corning): comprised of 24 μl enzyme, 1 μl compound, and 25 μl of cyclic nucleotide. The general assay buffer consists of 50 mM Tris-HCl (pH 7.5) and 0.1% (w/v) bovine serum albumin (Fatty Acid Free, Sigma-Aldrich). Enzyme concentrations, substrate concentrations, and various PDE-specific buffer additives are described in Supplementary Table 1. Compounds to be tested are diluted in pure dimethyl sulfoxide (DMSO) using 10-point concentration-response curves, and 1 μl is acoustically dispensed into assay plates using the Echo555 (LabCyte). Maximal compound concentration in the reaction mixture is either 10 or 100 μM. Compounds at the appropriate concentration are pre-incubated with either of the PDE enzymes for 30 min before the reaction is started by the addition of substrate ([8-^3^H]-cAMP, 20.7 Ci/mmol or [8-^3^H]-cGMP; 6.5 Ci/mmol, Perkin Elmer). Reactions are allowed to proceed for 60 min at room temperature before quenching. Next, reactions are stopped by the addition of 400 μg/per well SPA beads (RPNQ0150, Perkin Elmer) and a potent non-selective PDE inhibitor to quench the reaction. Bead bound radioactivity (product) is quantified 12 h later a Microbeta counter (Perkin Elmer). Data is normalized to % inhibition using standard methods (Campbell et al., 2004), and IC_50_ values were plotted using PRISM (GraphPad Software) using the 4 parameter logistic equation (Campbell et al., 2004). Data for potency values are expressed as the geometric mean and arithmetic standard deviation.

### Rat Telemetry Studies

Male Sprague Dawley (Taconic Biosciences) rats, Spontaneous Hypertensive rats (Charles River Laboratories, United States), and Dahl Salt Sensitive rats (Medical College of Wisconsin) were implanted with transmitters (HD S10; Data Science International) for collection of BP and heart rate (HR) data. The transmitters are capable of transmitting a BP signal via a pressure catheter inserted into the abdominal aorta. Initially, a baseline was established and recorded. Each rat was then removed from its recording cage and was dosed by p.o. gavage with vehicle or compound and the animals were then returned to the recording cage. Data sampling for BP and HR was carried out in intervals at selected time points for the duration of the experiment.

### Rat Ear Temperature

Male Sprague Dawley rats at 6 to 7 weeks were dosed orally by p.o. gavage with different doses of BTTQ formulated in 1% hydroxyethlycellulose (Dow Corning, Midland, MI, United States) (w/v), 0.25% polysorbate 80 (Sigma-Aldrich, St. Louis, MO, United States) (v/v), and 0.05% antifoam (v/v; Dow Corning, Midland, MI, United States) in purified water. Following dosing, ear temperature was measured by using a k-type thermocouple probe digital thermometer (Cole Parmer) every hour for 6 h and then at 24 h. Blood samples for plasma exposure measurement were obtained at the same time. Blood was collected via a tail snip directly into a 20-μL EDTA-coated capillary and immediately spotted onto a Whatman DMPK-C DBS card (GE Healthcare Bio-Sciences). Tail snips were performed by removing approximately 1 mm of the tail by using a scalpel. Blood flow was initiated by gentle squeezing of the tail. No analgesia or anesthesia was used during blood collections. A single sample and spot were collected per time point. The DBS cards were allowed to dry for approximately 2 h at room temperature, after which the cards were placed in a zip-top bag, stored, and shipped at ambient temperature.

### Studies in Isolated Vascular Segments (Myography Study)

For studies of the mesenteric arteries, rats were anesthetized using 5% isoflurane gas followed by euthanasia by secondary method. Small mesenteric arteries (∼300-μm internal diameter, 350-μm external diameter) supplying the small intestine were carefully excised under a dissecting microscope (Leica; Buffalo, NY, United States) and immersed in a cold physiological salt solution having the following ionic composition (in mM): NaCl (119.0), KCl (4.7), CaCl_2_ (1.6), NaH_2_PO_4_ (1.18), MgSO_4_ (1.17), NaHCO_3_ (24.0), D-glucose (5.5), and EDTA (0.03). The arteries were cannulated at the proximal and distal ends using glass micropipettes. The vessels were equilibrated with a 21% O_2_, 5% CO_2_, 74% N_2_ gas mixture at 37°C in the physiological salt solution for 30 min. The intraluminal pressure was maintained at 80 mmHg to approximate *in vivo* conditions, and the internal diameter of the artery was measured using television microscopy. Responses to vasodilator stimuli (different BTTQ concentrations) were determined in arteries precontracted with 10 μM phenylephrine (PE) and expressed as % increase from basal (PE-precontracted) diameter. Responses of the mesenteric arteries to the endothelium-dependent vasodilator agonist acetylcholine (ACh) were evaluated in SS rats fed a high salt diet (4% NaCl) for 3 weeks and treated with BTTQ (3 mg/kg) p.o. gavage twice daily or vehicle.

### Western Blot

Heart, aorta, and mesenteric artery samples collected from SS rats were pulse sonicated in Laemmli buffer with a protease inhibitor cocktail (Roche, Basel, Switzerland). The homogenates were centrifuged at 1000 *g* for 15 min at +4°C and sediment was discarded. The resulting supernatant was separated on SDS-PAGE, then transferred onto nitrocellulose membrane (Millipore, Bedford, MA, United States) and probed with PDE1 antibody (1:500, Thermoscintific Fisher, Invitrogen, Cat# PA5-87915), and subsequently visualized by enhanced chemiluminescence (Amersham Biosciences, Inc., Piscataway, NJ, United States).

### Statistical Analysis

All data in this manuscript were presented as mean ± SEM with a significance level of *p* < 0.05, and n represents the number of individual animals. The significance of differences in concentration-response curves in the ear temperature and, myograft study were calculated using *t*-test, compare to the vehicle group. The EC_50_ was calculated using GraphPad Prism 8.3 using a 4-parameter logistic non-linear curve fit model. Single-dose rat telemetry data for normal and SHR rats and repeat-dose telemetry data in Dahls salt-sensitive rats were analyzed by two-way repeated measures ANOVA. Treatment group main effects were compared by Tukey’s HSD method. All telemetry data were analyzed with GraphPad Prism 8.3.

## Results

### Identification of a Selective PDE1 Inhibitor

**FIGURE 1:**
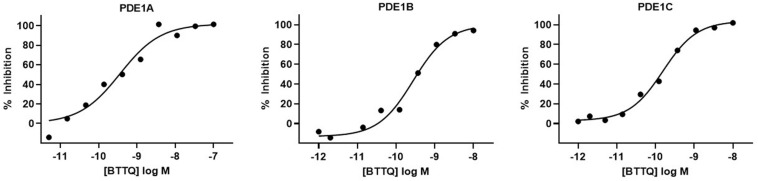
Inhibition of PDE1 enzyme activity by BTTQ. The compound was pre-incubated with different recombinant PDE1 isoforms for 30 min before the addition of the substrate. After incubating at room temperature for 60 min, reactions were stopped by the addition of SPA beads. cAMP level was measured after 12 h. IC_50_ values of PDE1A **(A)**, PDE1B **(B)**, and PDE1C **(C)** were calculated by plotting the normalized data vs. log [compound] and fitting the data using a four-parameter logistic equation.

In effort to generate a selective PDE1 inhibitor we synthesized the compound 1-[1,1′-**b**i(cyclopropyl)-1-yl]-5-(cyclopropylmethyl)-6,7,8,9-**t**etrahydro[1,2,4]**t**riazolo[4,3-A]**q**uinoxaline-4(5H)-one that we refer to hereafter as BTTQ. We tested BTTQ against all human and some rat recombinant PDE enzymes using a radioactivity-based enzyme activity assay. BTTQ inhibited all three isoforms of PDE1 with very comparable half-maximal inhibitory concentrations (IC_50_) (1, 0.5, and 0.2 nM for PDE1A, PDE1B, and PDE1C, respectively; Figures 1A–C), while other PDEs were only affected at high μM concentrations (Table 1). To ensure that BTTQ retains inhibition against rat PDE enzymes, we also tested rat PDE1B and PDE3A, which had respective IC_50_ values of 1 nM and inactive.

**TABLE 1:** Inhibition of PDE enzyme activity by BTTQ.

**BTTQ**	**IC_50_ nM (SD, *n*)**
Human PDE enzymes	
PDE1A	1.0 (0.8, *n* = 14)
PDE1B	0.45 (0.2, *n* = 4)
PDE1C	0.14 (0.3, *n* = 3)
PDE2A	>10,000 (*n* = 2)
PDE3A	>100,000 (*n* = 8)
PDE4D	12,000 (1000, *n* = 3)
PDE5A	>10,000 (*n* = 3)
PDE6A/6B	>10,000 (*n* = 4)
PDE7B	1600 (800, *n* = 2)
PDE8A	>10,000 (*n* = 2)
PDE9A	>10,000 (*n* = 2)
PDE10A	>10,000 (*n* = 2)
PDE11A	2,400 (800, *n* = 2)
Rat PDE enzymes	
PDE1B	1.0 (*n* = 1)
PDE3A	>100,000 (*n* = 1)

### Inhibition of PDE1 Caused Vasodilation in Normal Rats

**FIGURE 2:**
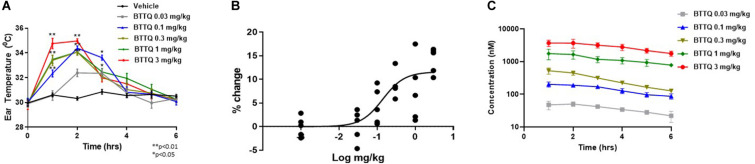
Effect of BTTQ on ear temperature in rats. Sprague Dawley rats were orally dosed with 0.03, 0.1, 0.3, 1, or 3 mg/kg of BTTQ and the temperature of the pinnae was measured with a K-probe thermometer every hour for 6 h after dosing. Ear temperature was plotted against time **(A)**, and median effective dose (ED_50_) was calculated using four parametric logistic equation during 1 h time point **(B)**. Data are given as mean ± SEM (*N* = 5). Data represent the mean of group average with *n* = 5 and the *p*-value calculated using one-way ANOVA compared to the vehicle group. **(C)** Pharmacokinetic profile of BTTQ. Plasma concentrations of BTTQ were determined after a single oral administration of 0.03, 0.1, 0.3, 1, and 3 mg/kg of the compound to Sprague Dawley rats. Data are given as mean ± SEM (*N* = 5). *p < 0.05, one way anova; **p < 0.005, one way anova.

To assess the role of PDE1 inhibition on vasodilation, normal Sprague Dawley (SD) rats were administered orally, a single dose of BTTQ at 0.03, 0.1, 0.3, 1, or 3 mg/kg and ear temperature was measured. The ear temperature method was used to test if vasodilation caused by PDE1 inhibition would increase the blood flow in the rat ear arteriole that would subsequently increase the surface temperature. Indeed, there was a significant rapid increase of ear temperature observed as high as 14% at the highest dose of 3 mg/kg within 1 h compared to the vehicle control, as shown in Figure 2A. A significant dose-dependent increase in ear temperature was observed at 0.1, 0.3, and 1 mg/kg. The dose-dependent increase in ear temperature stayed significant for up to 3 h and returned to baseline for all doses at 6 h. The pharmacokinetic analysis revealed a dose-dependent increase in plasma concentration of BTTQ that maintained for several hours after oral administration (Figure 2C and Supplementary Table 2) with a half-life of almost 4 h. The calculated percent target engagement based on the unbound drug concentration and with hill slope of 1 demonstrated about 65% target engagement (TE) at the lowest dose at 1 h and saturated after 0.3 mg/kg (Table 2). At this level of target engagement, the median effective dose (ED_50_) was 0.14 mg/kg as calculated using four parametric logistic equation (Figure 2B).

**TABLE 2:** Target engagement by BTTQ.

**Dose**	**IC_50_**	**Unbound compound concentration (nM)**	**% TE**
0.03	1.37	2.52	64.78
0.1		10.6	88.55
0.3		27.7	95.29
1		92.5	98.54
3		197	99.31

### PDE1 Inhibition Lowered Blood Pressure in Normal Sprague Dawley, Spontaneously Hypertensive, and Dahl Salt Sensitive Rats

**FIGURE 3:**
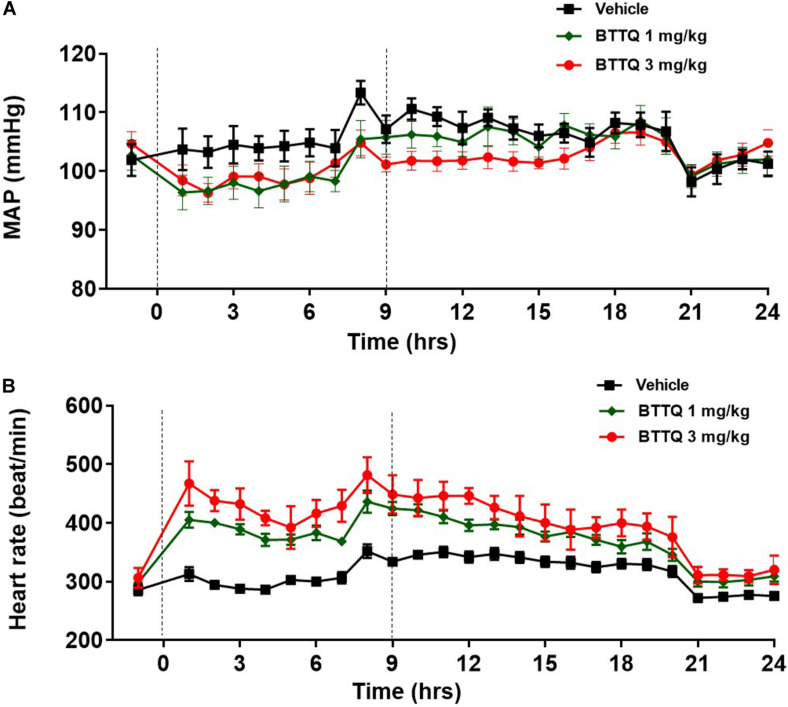
Effect of BTTQ on the blood pressure and heart rate of SD rats. SD rats were dosed twice daily, and **(A)** mean arterial pressure (MAP), and **(B)** heart rate were recorded for 24 h with telemetry. Dotted line represents the timing of doses. All the data represented as mean ± SEM. The main effect of BTTQ treated animals is significantly different from vehicle, *p* < 0.0001 for high dose group.

To evaluate the hemodynamic effect of PDE1 inhibition in normal animals, BTTQ was administered in freely moving Sprague Dawley (SD) rats. They were dosed at 1 and 3 mg/kg twice daily via oral gavage, and the data was captured every hour for 24 h with telemetry. As shown in Figure 3A, there was a significant reduction in the mean arterial pressure (MAP), with a concomitant increase in heart rate (Figure 3B) compared to the vehicle-treated group. An immediate decrease of about 5 mmHg in MAP has been observed in both groups within 1 h of the first dose compared to the vehicle. In general, the BP-lowering effect sustained longer in the high dose group and returned to baseline post 7 h of the second dose. A dose-dependent increase in heart rate was observed, which showed a trend to return to baseline within the time of measurement.

**FIGURE 4:**
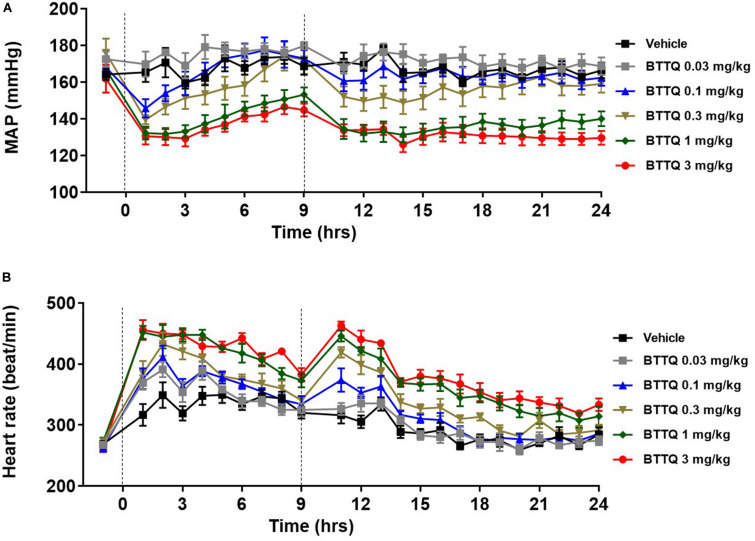
Effect of BTTQ on the blood pressure and heart rate of SHR rats. SHR rats were dosed twice daily, and **(A)** mean arterial pressure (MAP), and **(B)** heart rate were recorded for 24 h with telemetry. Dotted line represents the timing of doses. All the data represented as mean ± SEM. The main effect of BTTQ treated animals is significantly different from the vehicle, *p* < 0.0001 for high dose group.

The significant hemodynamic effect of PDE1 inhibition, as seen in the normal rats suggested that inhibition of PDE1 might be beneficial in the context of hypertension. In order to test this, SHR rats were dosed orally with 0.03, 0.1, 0.3, 1, and 3 mg/kg twice daily, and cardiovascular parameters were recorded for 24 h with telemetry. As shown in Figure 4A, a dose-dependent decrease in the MAP was observed. The lowest dose of 0.03 mg/kg did not show any effect in both BP and HR in SHR. The magnitude of BP-lowering effect was much greater in SHR rats compared to normal rats when compared at the same dose level. A dose-dependent increase in heart rate was also evident in SHR rats (Figure 4B) with a relatively greater magnitude compared to SD rats within the same dose level.

**FIGURE 5:**
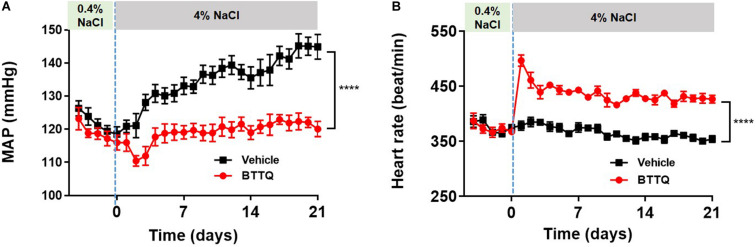
Effect of BTTQ on the blood pressure and heart rate of Dahl SS rats. SS rats were dosed twice daily with BTTQ (3 mg/kg) through oral gavage. **(A)** Mean arterial pressure (MAP) and **(B)** heart rate were recorded with telemetry for 5 days on a normal salt diet (0.4% NaCl) and 21 days after switching the animals to a high salt diet (4% NaCl). Data represent mean ± SEM. ****p-value < 0.0001.

To test the anti-hypertensive effect of the PDE1 inhibitor over a longer duration in a model of salt-induced hypertension, Dahl SS rats were dosed orally with BTTQ (3 mg/kg twice daily) for 21 days while keeping them on a high salt (4%) diet. There was a profound reduction in the MAP in the PDE1 inhibitor group compared to the vehicle-treated group (Figure 5A), which was accompanied by elevation of heart rate (Figure 5B).

**FIGURE 6:**
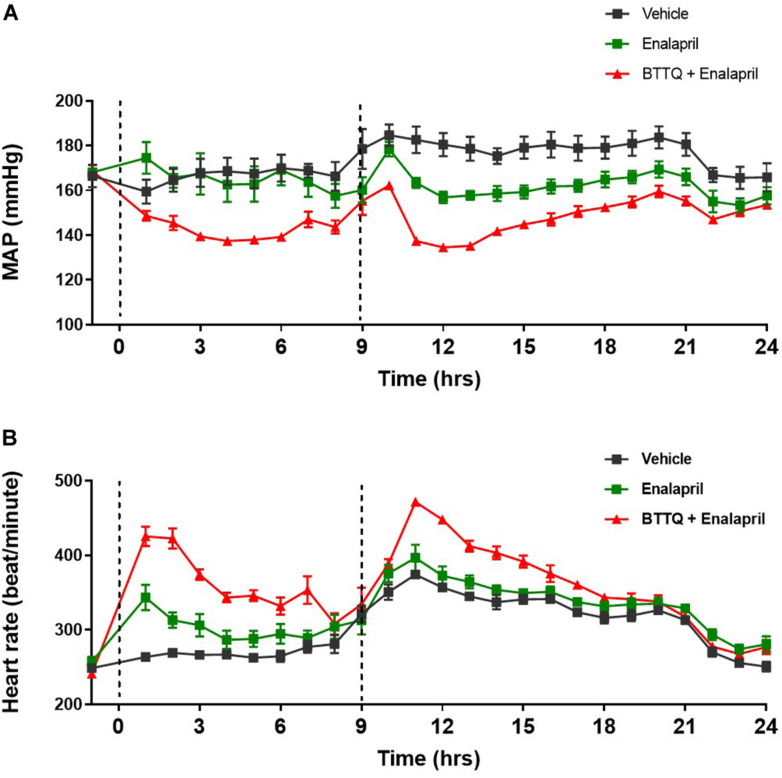
Effect of combined BTTQ and enalapril therapy on the blood pressure and heart rate of SHR rats. SHR rats were dosed twice daily, and **(A)** mean arterial pressure (MAP), and **(B)** heart rate were recorded for 24 h with telemetry. Dotted line represents the timing of doses. All the data represented as mean ± SEM. The main effect of BTTQ + Enalapril treated animals is significantly different from enalapril alone, *p* = 0.004.

In clinic, ACE inhibitors are used as a standard of care for hypertensive patients. In order to evaluate the additive effect of PDE1 inhibitor with ACE inhibitor, SHR rats were orally dosed (twice daily) either with 3 mg/kg enalapril alone or in combination with 0.3 mg/kg of BTTQ and cardiovascular parameters were recorded for 24 h. As expected, enalapril lowered the MAP by 13% without affecting the heart rate (Figure 6). The co-administration of PDE1 on top of enalapril similarly lowered MAP by almost 30% (Figure 6A) with a significant increase in heart rate, which kept elevated for a long period of time (Figure 6B). Effects of BTTQ on systolic and diastolic blood pressure in SD, SHR, and Dahl SS rats are shown on Supplementary Figures 1–4.

### Myography Studies in Isolated Vascular Segments

**FIGURE 7:**
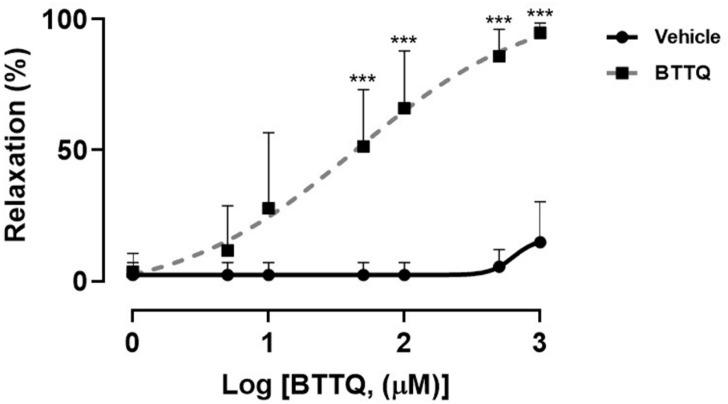
Concentration-response curve showing the percent of relaxation of pre-constricted third-order mesenteric artery from Dahl SS rats that were on a normal diet (0.4% NaCl) in response to varying concentrations of BTTQ. Data represent mean ± SEM. ***p-value < 0.001.

The expression of PDE1 in the wall of mesenteric artery isolated from SS rat, was confirmed using western blot technique which also revealed the abundance of PDE1 in other parts of the cardiovascular system, i.e., heart, and aorta (Supplementary Figure 5). The third-order mesenteric arteries from Dahl SS rats were constricted at the beginning of the experiment using 10 μM of phenylephrine, then escalating doses of PDE1 inhibitor or vehicle were added, and the inner diameter was measured after stabilization. Experiments reveal concentration-dependent relaxation in mesenteric arteries with PDE1 inhibitor compared to the vehicle treatment that reached about 92% of the initial fully relaxed diameter with 1 mM. On the contrary, the vehicle did not show a significant relaxing effect (Figure 7). The same set up was used to test the effect of serial concentration of acetylcholine to induce vasorelaxation on pre-constricted mesenteric artery using 10 μM of phenylephrine from Dahl SS rats that have treated through oral gavage with PDE1 inhibitor and kept on a high salt diet for 21 days, however, no significant difference between the two groups was observed (Supplementary Figure 6).

## Discussion

In this manuscript, we presented a novel, potent PDE1 inhibitor BTTQ highly selective for PDE1 over all other PDES but equipotent for all isoforms of PDE1 (A, B, and C). This compound possesses ADME characteristics allowing its use *in vivo*. BTTQ demonstrated BP-lowering effects in both normotensive rats and animals with hypertension of different genesis (SHR rat, a high renin model, and Dahl SS rat, a low renin model of salt-induced hypertension) suggesting the pattern of activity independent of primary mechanisms of hypertension. Moreover, additional BP lowering could be achieved when BTTQ was administered on top of ACE inhibitor lisinopril, a current standard of care in the treatment of hypertension. These anti-hypertensive effects are likely associated with vasodilatory properties of BTTQ that we have demonstrated *in vitro*, using isolated mesenteric arteries, and *in vivo*, using novel Pk/Pd model. Administration of BTTQ was associated with increased heart rate in both models of hypertension as well as in the normotensive rats.

PDE1 attracted interest in the context of arterial hypertension when an association of PDE1A single nucleotide polymorphism with diastolic and mean blood pressure was described in human genetics study (Tragante et al., 2014). Importantly, this association was further confirmed by a different research group in an independent cohort of patients (Bautista Nino et al., 2015). However, mechanistic experimental studies lagged behind due to the lack of potent and selective PDE1 inhibitors suitable for *in vivo* use in the animal models of disease. In a seminal conceptual study, Giachini et al. (2011) postulated the role of PDE1 in Angiotensin II-induced hypertension and vascular contraction. In this study, the authors used vinprocetin, a natural product with low potency and poor selectivity. Only relatively recently, Khammy et al. (2017) followed up with confirmatory data supported by potent and selective PDE1 inhibitors. Based on PDE gene expression data and using compounds with different affinity to PDE1 isoforms, they concluded that PDE1A inhibition was primarily responsible for this pharmacological effect.

Our current data obtained using a structurally unrelated compound further supports the critical involvement of PDE1 into the regulation of blood pressure via demonstration of therapeutic benefits in two independent rodent models of hypertension. While SHR rats share important mechanistic features with Angiotensin II infusion model, it is, arguably, more representative of human disease (Doggrell and Brown, 1998). Another animal model used in the current investigation, Dahl salt-sensitive hypertension, capitalizes on different mechanisms of blood pressure regulation (Rapp, 1982) and commonly used to study salt-induced hypertension (Palygin et al., 2017; Fehrenbach et al., 2019; Lerman et al., 2019). Our current data obtained using a structurally unrelated compound further supports the critical involvement of PDE1 into the regulation of blood pressure via demonstration of therapeutic benefits in two independent rodent models of hypertension. While SHR rats share important mechanistic features with Angiotensin II infusion model, it is, arguably, more representative of human disease (Doggrell and Brown, 1998). Another animal model used in the current investigation, Dahl salt-sensitive hypertension, capitalizes on different mechanisms of blood pressure regulation (Rapp, 1982) and commonly used to study salt-induced hypertension (Palygin et al., 2017; Fehrenbach et al., 2019; Lerman et al., 2019). Several studies have shown expression of PDE1 in the vascular wall (e.g., aorta, femoral, and mesenteric artery) of different rat strains, e.g., Wistar (Khammy et al., 2017; Laursen et al., 2017), Sprague Dawley (Giachini et al., 2011), and SHR rats (McMahon et al., 1989; Pontes et al., 2012). We also have confirmed the expression of the PDE1 in the mesenteric artery as well as other parts in the cardiovascular system (i.e., heart and aorta) of Dahl salt sensitive rats using western blot (Supplementary Figure 5). The fact that BTTQ was equally efficacious in both models, suggesting that PDE1 inhibition acts at the downstream signaling common for BP control regardless of the factors that initiated hypertension.

PDE1 is likely to be involved in multiple mechanisms regulating BP. Our data strongly suggest that peripheral vasodilation could play a critical role. First, we demonstrated direct dose-dependent relaxation of pre-constricted mesenteric arteries. Second, BTTQ increased rat ear temperature in a dose-dependent manner. This change in temperature is likely a result of increased local blood flow driven by vasodilation. Importantly, plasma levels of the compound and, hence, tissue exposure, were comparable with compound concentrations *in vitro* that induced vasodilation and exceeded the IC_50_ for PDE1A. Given that diameter of small arteries and arterioles is directly related to peripheral vascular resistance, and the latter, together with the pumping capacity of the heart and blood volume, ultimately defines BP (Mayet and Hughes, 2003), we surmise that vasodilation could be the main mechanism of the anti-hypertensive effects of PDE1 inhibitors.

Since PDE1A is the major isoform expressed in arterial SMC (Giachini et al., 2011; Khammy et al., 2017), it is highly likely that BP lowering activity of BTTQ is primarily driven by its inhibition of PDE1A. The preferred substrate of PDE1A is cGMP that is produced in vascular SMCs by nitric oxide-induced soluble guanylate cyclase or natriuretic peptide-activated particulate guanylate cyclase (Francis et al., 2010). Downstream, cGMP signaling is associated with activation of protein kinase G, ultimately leading to SMC relaxation. When PDE1 is activated, it degrades cGMP and hence impedes vasorelaxation. Interestingly, PDE1 is the only PDE activated by calcium (Maurice et al., 2014). Calcium signaling is enhanced in arterial hypertension (Pinterova et al., 2011). Therefore, it is reasonable to suggest that PDE1A activation serves as one of the major mechanisms associated with increased peripheral vascular resistance, and its inhibition would have significant therapeutic benefit. Importantly, the potential role of PDE1 in the development of Autosomal Dominant Polycystic Kidney Disease (ADPKD) was shown. It was reported that calcium- and calmodulin-dependent PDEs (PDE1A and PDE1C) and PDE3A modulate the development of PKD, possibly through the regulation of compartmentalized cAMP pools that control cell proliferation and CFTR-driven fluid secretion (Ye et al., 2016). Pde1a mutant mice had a mild renal cystic disease and a urine concentrating defect on a wild-type genetic background and aggravated renal cystic disease on a Pkd2^WS25/–^ background (Wang et al., 2017). Based on these observations it was proposed that PDE1A plays an important role in the renal pathogenesis of ADPKD and in the regulation of BP.

PDE1 inhibition, however, could also be responsible for an observed increase in heart rate. Tachycardia is not unique to BTTQ. It is likely to be the class effect since structurally unrelated PDE1 inhibitors increased heart rate in different species of experimental animals (Hashimoto et al., 2018). The precise mechanism of PDE1-induced tachycardia needs to be further investigated. However, it likely has both extracardiac and intracardiac components. The former could be a result of baroreflex, activation of sympathetic signaling induced by BP lowering (Lanfranchi and Somers, 2002) that, to some degree, is germane to any peripheral vasodilator (Pettinger and Mitchell, 1988). The latter is likely to be specifically associated with inhibition of PDE1A. Lukyanenko et al. (2016) reported that PDE1 is expressed in the rabbit sinoatrial node. While cGMP is a preferred substrate of this enzyme, PDE1 is also capable of binding to (albeit with lower affinity) and degrading cAMP. cAMP, in turn, can stimulate calcium signaling resulting in increased pacemaker activity (Lukyanenko et al., 2016). It is still unknown whether this mechanism is involved in tachycardia in the rat, and, most critically, is translatable to the human.

In general, cross-species translation remains an issue given a significant difference in the patterns of PDE1 expression in the heart. Recent studies by Hashimoto et al. (2018) demonstrated that PDE1 inhibition with ITI-214, a PDE1 inhibitor with similar potency and selectivity (Li et al., 2016), was simultaneously associated with positive inotropic effects (most likely driven by inhibition of PDE1C in cardiomyocytes) and peripheral vasodilation (most likely driven by inhibition of PDE1A in vascular SMCs). This “ino-dilation” provides intriguing prospects for PDE1 inhibitors in patients with heart failure. ITI-214 has recently completed the second dose cohort (single dose of 30 mg administered orally) a proof-of-mechanism study in patients with chronic heart failure (ClinicalTrials.gov Identifier: NCT03387215). In addition, ITI-214 has also finished Phase II clinical trial for Parkinson’s disease (ClinicalTrials.gov Identifier: NCT03257046).

Despite significant improvement in patient care, resistant arterial hypertension remains an unmet medical need (Hashimoto et al., 2018). Blood pressure-lowering effects of PDE1 inhibitor BTTQ in two mechanistically distinct animal models of hypertension and activity on top of standard of care, demonstrated in the current study, suggest a potential benefit of PDE1 inhibitors for this patient population. Vasodilatory properties/reduction of peripheral vascular resistance could also be instrumental for the treatment of other diseases beyond arterial hypertension (chronic kidney disease, improvement of brain circulation, etc.).

## Data Availability Statement

All datasets generated for this study are included in the article/Supplementary Material.

## Ethics Statement

The animal study was reviewed and approved by Eli Lilly and Company’s Animal Care and Use Committees or by IACUC at the Medical College of Wisconsin.

## Author Contributions

AD and SK designed the study, interpreted and analyzed the data, and drafted the manuscript. JB, LP, XZ, MT, and TM performed the experiments and made the analyses. JH reviewed the statistical analysis of the data. MR, FW, AS, MK, and SA revisited it critically for important intellectual content design and manuscript preparation. CJ, KR, and MW contributed to the chemical synthesis of the BTTQ. MR conceived the project. All authors provided approval for the publication of the final manuscript.

## Conflict of Interest

MR, JB, CJ, TM, and MW were employed by the company Eli Lilly and Company.

The remaining authors declare that the research was conducted in the absence of any commercial or financial relationships that could be construed as a potential conflict of interest.
