# Supplementary material for: Selective Phosphodiesterase 1 Inhibitor BTTQ Reduces Blood Pressure in Spontaneously Hypertensive and Dahl Salt Sensitive Rats: Role of Peripheral Vasodilation
Source: Front Physiol. 2020 Sep 8;11:543727. doi: 10.3389/fphys.2020.543727 (PMC7506137; doi:10.3389/fphys.2020.543727)
Supplement: Supplementary file 4 [file Presentation_1.PPTX]

## Slide 1
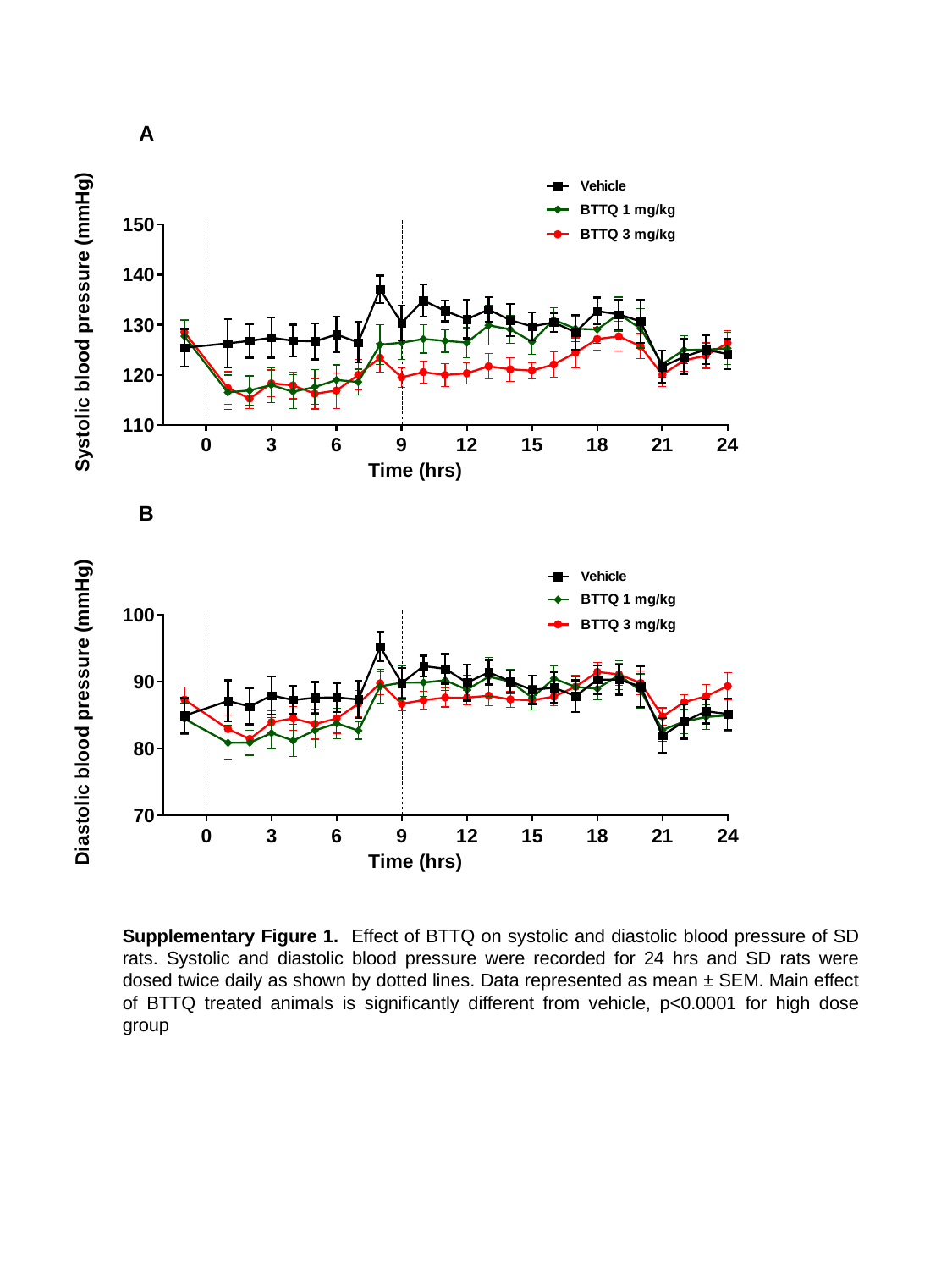

A
B
Supplementary Figure 1. Effect of BTTQ on systolic and diastolic blood pressure of SD rats. Systolic and diastolic blood pressure were recorded for 24 hrs and SD rats were dosed twice daily as shown by dotted lines. Data represented as mean ± SEM. Main effect of BTTQ treated animals is significantly different from vehicle, p<0.0001 for high dose group

## Slide 2
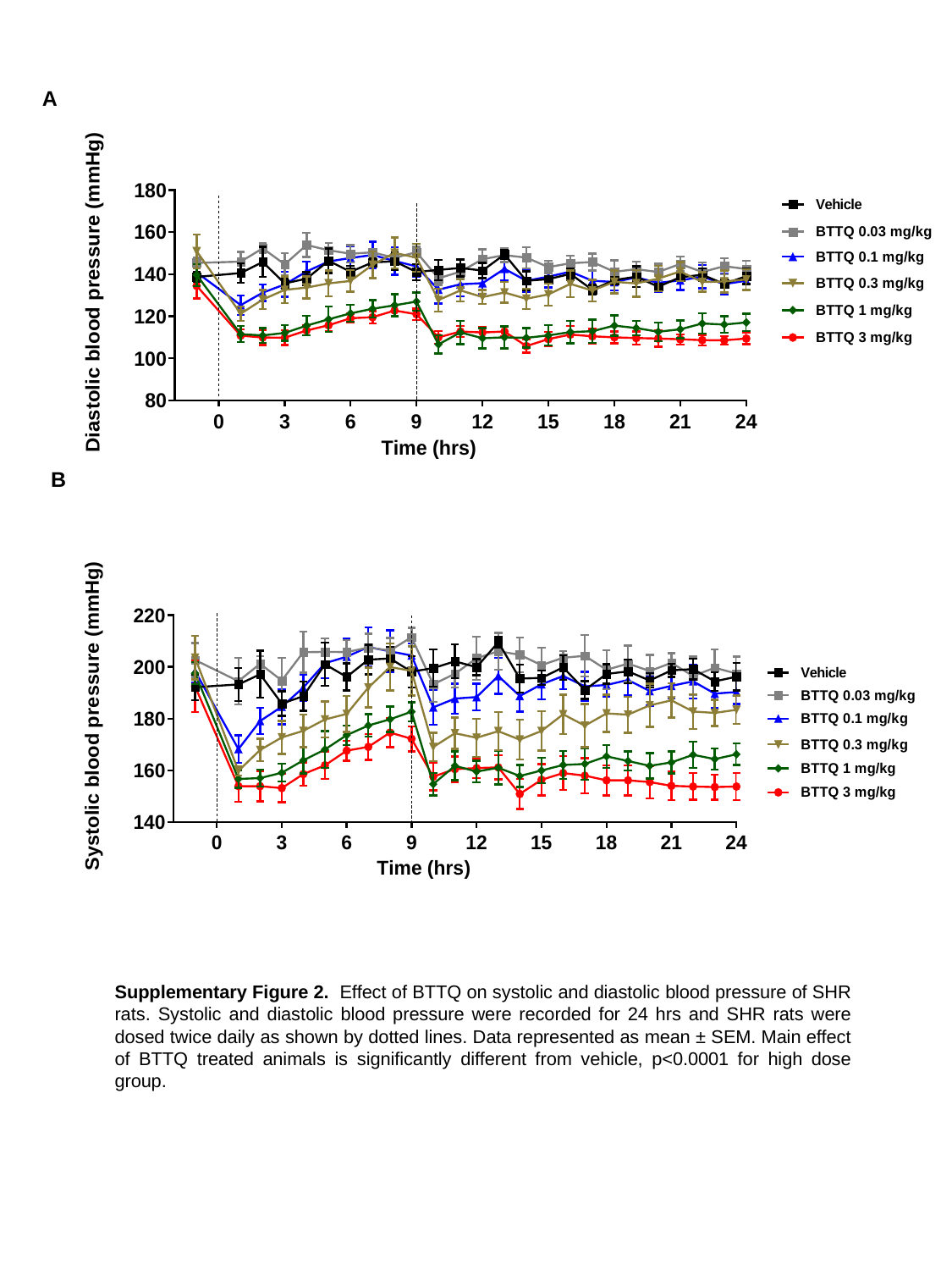

A
B
Supplementary Figure 2. Effect of BTTQ on systolic and diastolic blood pressure of SHR rats. Systolic and diastolic blood pressure were recorded for 24 hrs and SHR rats were dosed twice daily as shown by dotted lines. Data represented as mean ± SEM. Main effect of BTTQ treated animals is significantly different from vehicle, p<0.0001 for high dose group.

## Slide 3
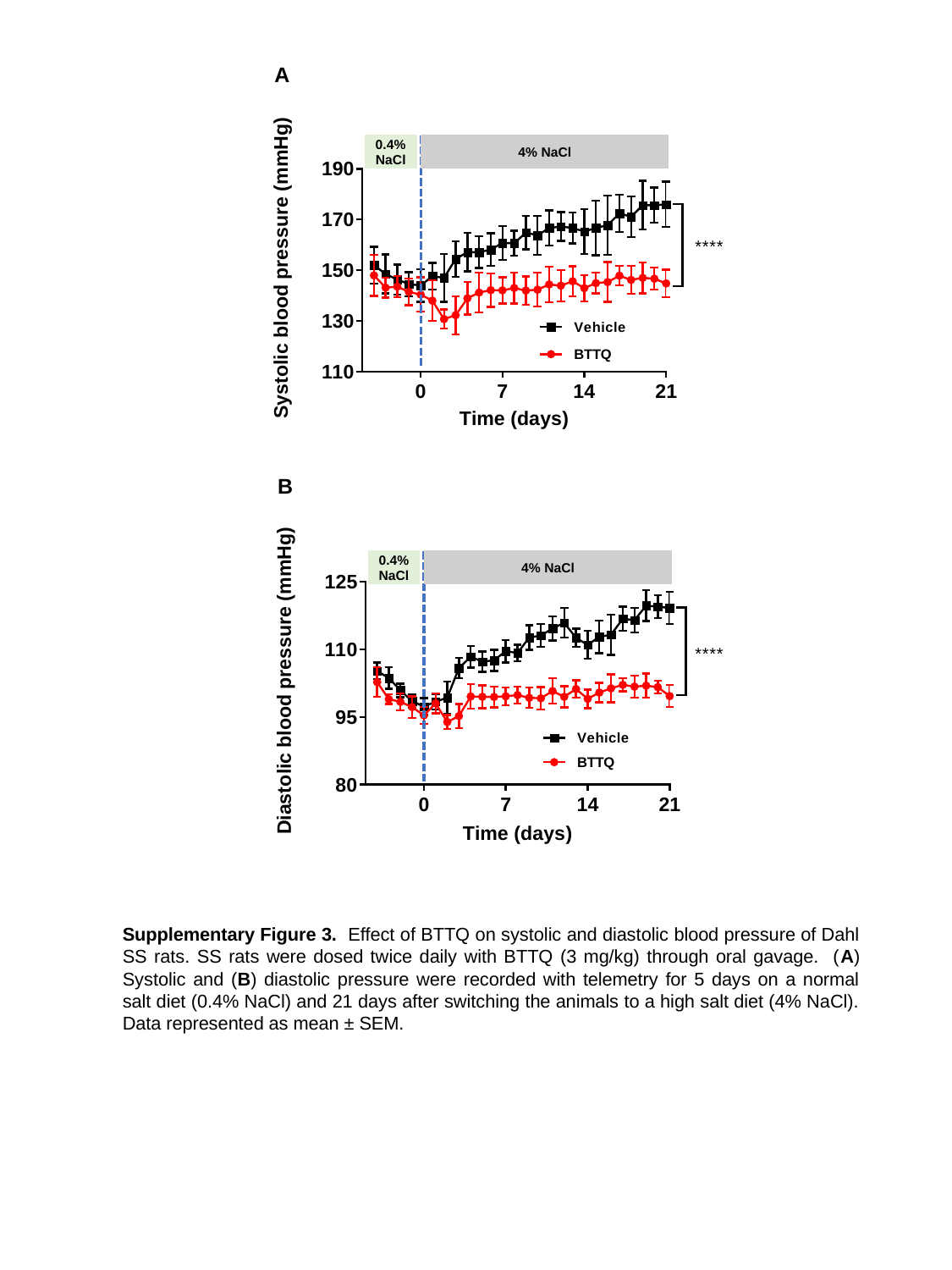

A
0.4% NaCl
4% NaCl
B
0.4% NaCl
4% NaCl
Supplementary Figure 3. Effect of BTTQ on systolic and diastolic blood pressure of Dahl SS rats. SS rats were dosed twice daily with BTTQ (3 mg/kg) through oral gavage. (A) Systolic and (B) diastolic pressure were recorded with telemetry for 5 days on a normal salt diet (0.4% NaCl) and 21 days after switching the animals to a high salt diet (4% NaCl). Data represented as mean ± SEM.

## Slide 4
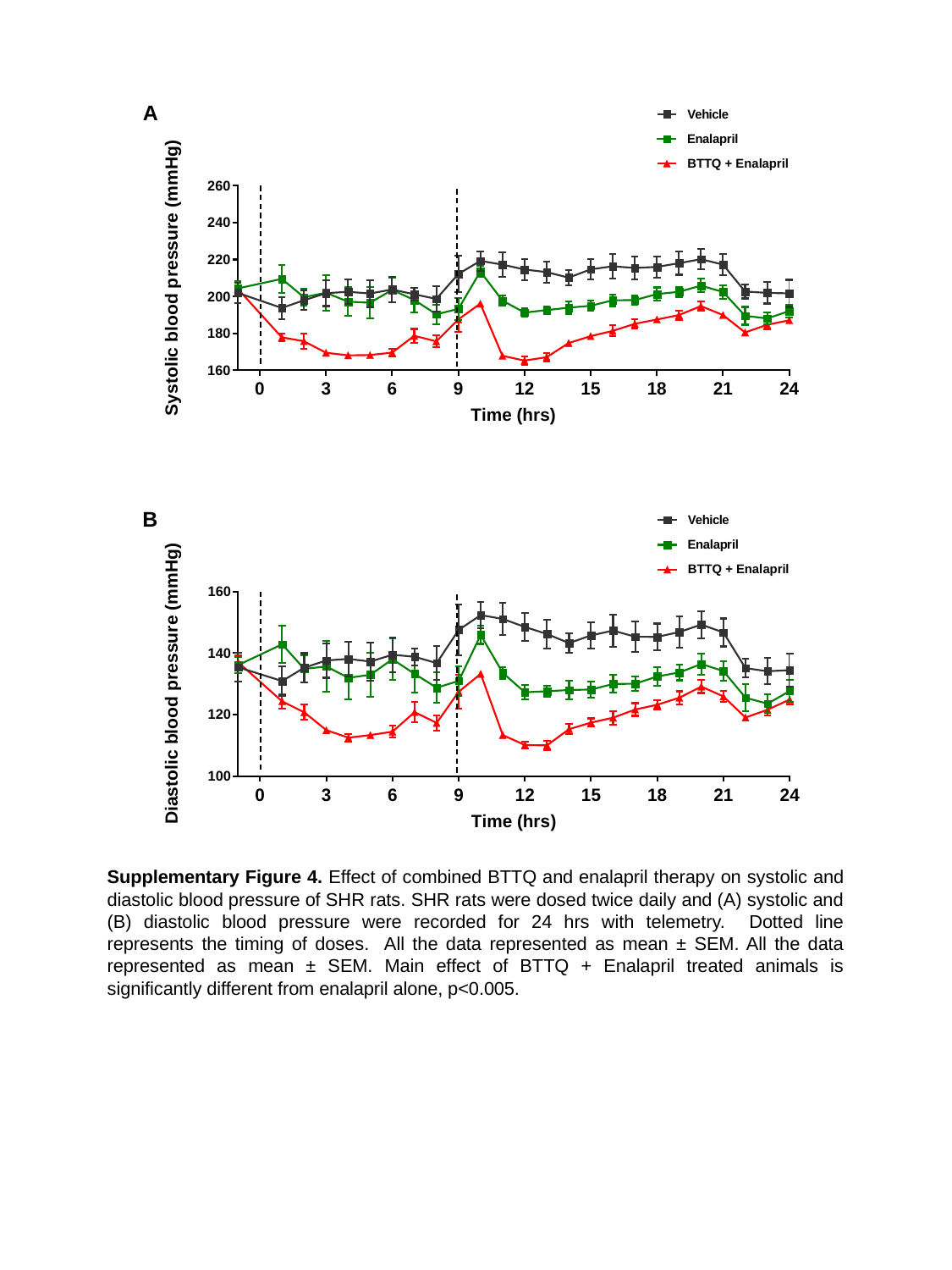

A
B
Supplementary Figure 4. Effect of combined BTTQ and enalapril therapy on systolic and diastolic blood pressure of SHR rats. SHR rats were dosed twice daily and (A) systolic and (B) diastolic blood pressure were recorded for 24 hrs with telemetry. Dotted line represents the timing of doses. All the data represented as mean ± SEM. All the data represented as mean ± SEM. Main effect of BTTQ + Enalapril treated animals is significantly different from enalapril alone, p<0.005.

## Slide 5
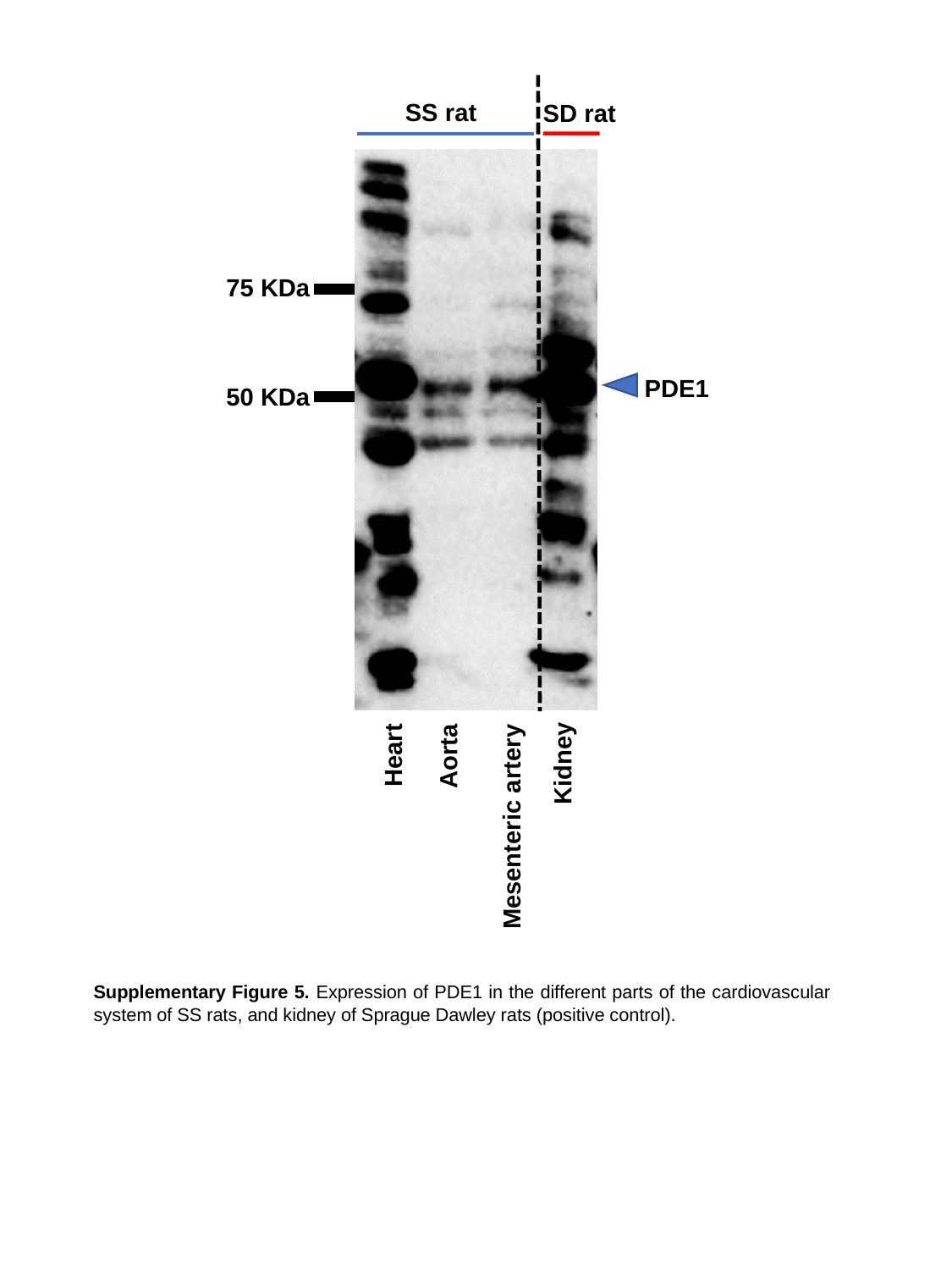

SS rat
SD rat
75 KDa
PDE1
50 KDa
Kidney
Aorta
Heart
Mesenteric artery
Supplementary Figure 5. Expression of PDE1 in the different parts of the cardiovascular system of SS rats, and kidney of Sprague Dawley rats (positive control).

## Slide 6
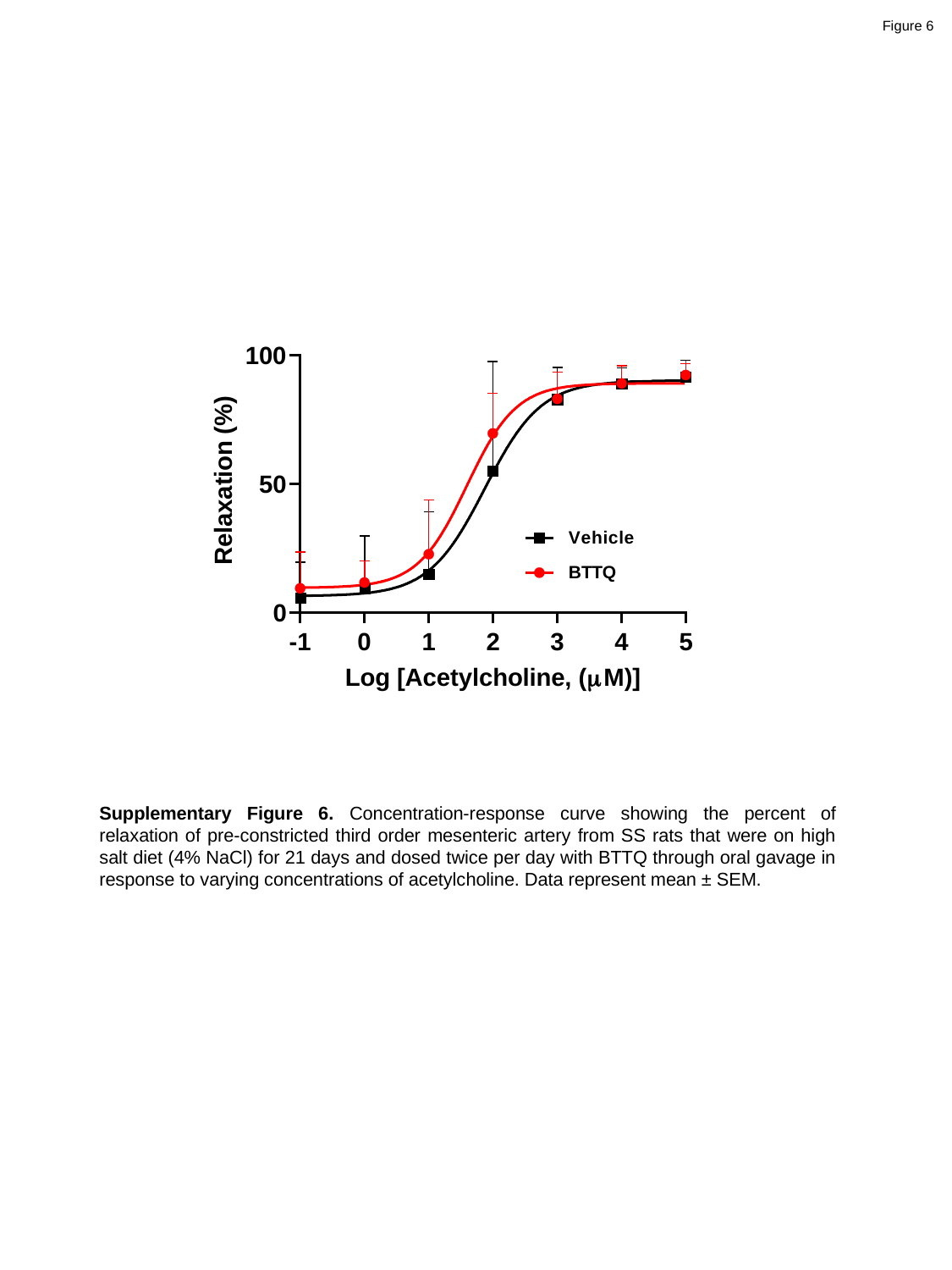

Figure 6
Supplementary Figure 6. Concentration-response curve showing the percent of relaxation of pre-constricted third order mesenteric artery from SS rats that were on high salt diet (4% NaCl) for 21 days and dosed twice per day with BTTQ through oral gavage in response to varying concentrations of acetylcholine. Data represent mean ± SEM.
